# Auditory cues for somatosensory targets invoke visuomotor transformations: Behavioral and electrophysiological evidence

**DOI:** 10.1371/journal.pone.0215518

**Published:** 2019-05-02

**Authors:** Gerome A. Manson, Luc Tremblay, Nicolas Lebar, John de Grosbois, Laurence Mouchnino, Jean Blouin

**Affiliations:** 1 Aix-Marseille University, CNRS, LNC FR 3C, Marseille, France; 2 University of Toronto, Centre for Motor Control, Faculty of Kinesiology and Physical Education, Toronto, Ontario, Canada; University of Ontario Institute of Technology, CANADA

## Abstract

Prior to goal-directed actions, somatosensory target positions can be localized using either an exteroceptive or an interoceptive body representation. The goal of the present study was to investigate if the body representation selected to plan reaches to somatosensory targets is influenced by the sensory modality of the cue indicating the target’s location. In the first experiment, participants reached to somatosensory targets prompted by either an auditory or a vibrotactile cue. As a baseline condition, participants also performed reaches to visual targets prompted by an auditory cue. Gaze-dependent reaching errors were measured to determine the contribution of the exteroceptive representation to motor planning processes. The results showed that reaches to both auditory-cued somatosensory targets and auditory-cued visual targets exhibited larger gaze-dependent reaching errors than reaches to vibrotactile-cued somatosensory targets. Thus, an exteroceptive body representation was likely used to plan reaches to auditory-cued somatosensory targets but not to vibrotactile-cued somatosensory targets. The second experiment examined the influence of using an exteroceptive body representation to plan movements to somatosensory targets on pre-movement neural activations. Cortical responses to a task-irrelevant visual flash were measured as participants planned movements to either auditory-cued somatosensory or auditory-cued visual targets. Larger responses (i.e., visual-evoked potentials) were found when participants planned movements to somatosensory vs. visual targets, and source analyses revealed that these activities were localized to the left occipital and left posterior parietal areas. These results suggest that visual and visuomotor processing networks were more engaged when using the exteroceptive body representation to plan movements to somatosensory targets, than when planning movements to external visual targets.

## Introduction

In a game of “Simon Says”, spoken instructions such as “touch your elbow” can prompt movements towards a specified body location. Similarly, the feeling of a mosquito landing on the elbow could also prompt movements to this same position. Although the goal of both actions is to reach to a common body location (i.e., hereafter referred to as a somatosensory target), the manner in which this position is identified could influence the body representation used to determine the target’s coordinates. The purpose of the present study was to examine if the modality of the stimulus indicating a somatosensory target’s position influences the body representation used to plan movements.

Evidence that movements to somatosensory targets could be planned using multiple representations is drawn from studies of autotopagnosia, a rare nervous system disorder characterized by the inability to localize and orient one’s own body parts [1–3]. In a case study by Sirigu et al. [4], a patient with autotopagnosia was tested on her ability to point to her own body positions in response to different instructional cues. The authors found that the patient was inaccurate when she pointed to the target body positions in response to verbal instructions but was accurate when she was instructed to point to the felt location of small objects placed on these same target body positions (see also: [5]). Based on these findings, the authors hypothesized that an exteroceptive, visually-based, body representation was used to plan movements in response to verbal cues, whereas an interoceptive, somatosensory-based, body representation was used to plan movements in response to tactile cues (see [6] for a review of different body representation taxonomies; and [7] and [8, 9] for additional theoretical discussions).

It is difficult to determine if the use of different body representations in patients with autotopagnosia emerges as an adaptation to their neurological impairment, or if this context-dependent selection of a body representation for movement planning also occurs in healthy individuals. In the present study, we sought to examine the contribution of the exteroceptive and interoceptive body representations to sensorimotor transformations during motor planning in healthy individuals by evaluating reaching movements towards somatosensory targets cued by simple auditory and vibrotactile stimuli.

In Experiment 1, to examine the body representation and sensorimotor transformations used to prepare movements to somatosensory targets, we asked participants to reach to somatosensory targets while looking at either a central fixation point or a peripheral fixation point (i.e., a gaze-shifted trial; see: [10–16]). Reaching endpoint errors from gaze-shifted trials were contrasted with reaching endpoint errors from central fixation trials to compute the bias induced by the gaze shift. The rationale for using this experimental paradigm is that errors increase in magnitude with peripheral fixation eccentricity when an exteroceptive, visually-based, representation is used to plan movements (i.e., gaze-dependent coding). Movements are classified as gaze-dependent if reaching endpoint errors are biased in the opposite direction of the gaze shift. This increase in gaze-dependent reaching endpoint error is likely the result of the magnification factor in perifoveal region [10]. Conversely, movements are classified as gaze-independent if the peripheral gaze-shift does not produce significant increases in reaching endpoint error. If reaching endpoint errors are not influenced by the gaze-shift, movement planning was likely performed using an interoceptive, somatosensory-based, representation [13]. Previous studies have demonstrated that movements to visual targets are planned using gaze-dependent representations [see 10, 12, 15, 17]. In contrast, studies examining movement planning to somatosensory targets have revealed that both gaze-dependent and gaze-independent representations may be used [13–15, 17].

Although there is evidence for flexibility in the encoding of somatosensory targets [13, 14], it is not clear if the cue used to indicate a somatosensory target’s location influences the body representation and sensorimotor transformations used for motor planning. In the present study, we investigated the influence of the cue modality by directly comparing the effect of a gaze shift on movements to one of three possible somatosensory targets: either the index, middle, and ring finger of the non-reaching hand. Somatosensory targets were identified by either an exogenous auditory cue (i.e., intensities of sound indicating which finger to reach to) or a direct tactile stimulation. To provide a reference of gaze-dependent reaching errors, participants also performed movements to visual targets cued by an auditory stimulus.

## Experiment 1 methods

### Participants

Ten participants (8 female; mean age: 24.4 ± 2.6 yrs.; range: 21–28) volunteered for the experiment. All participants were right-handed with normal or corrected-to-normal vision. The experiment took 1.5 hours to complete. Informed consent was obtained prior to the beginning of the experiment and the local research ethics committee at Aix-Marseille University approved all procedures. The protocols and procedures employed were in accordance with the 1964 Declaration of Helsinki.

### Apparatus

**Fig 1:**
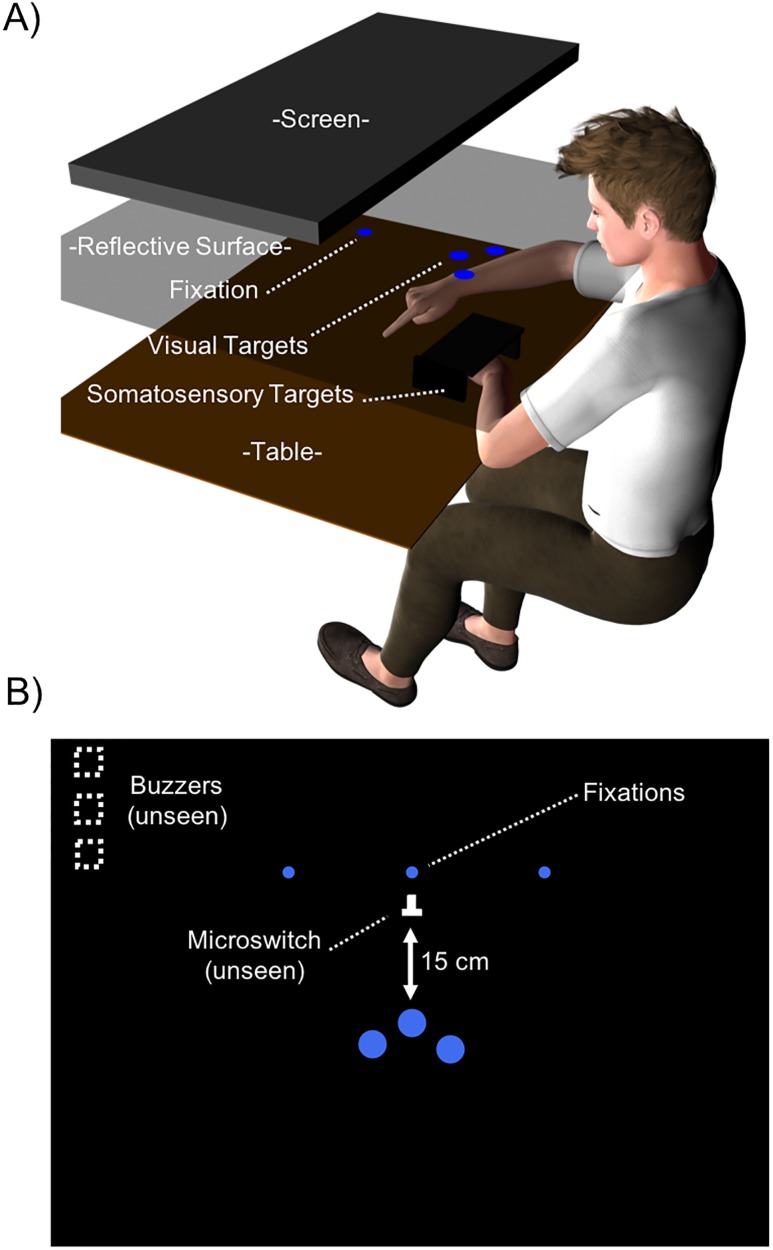
A) A depiction of the apparatus used in Experiment 1 (not to scale). Participants sat facing an immersive display comprised of a computer monitor, a semi-reflective glass surface, and a custom aiming apparatus. Participants made movements from an unseen home position (microswitch) to either visual targets projected onto the surface of the aiming console or the perceived position of one of their three middle fingers (i.e., the index, middle, ring) of their non-reaching hand (i.e., somatosensory target conditions). In the somatosensory target conditions, participants positioned their target fingers beneath a plastic case and performed movements to the perceived position of their fingers as if projected onto the case’s surface. Note that the visual items projected on the semi-reflective glass were perceived by the participants as appearing on the table surface. B) A depiction of the aiming surface in the visual target condition from the participant’s perspective (not to scale). Piezoelectric buzzers were positioned to the left of the aiming surface, and provided the imperative signals in the auditory-cued aiming conditions.

A depiction of the experimental setup is presented in Fig 1. The experiment took place in a dark room, where participants were seated comfortably in front of a custom-built aiming apparatus (see Fig 1B). Once seated, participants placed their forehead on a stabilizing headrest located above the aiming apparatus. From this position, participants viewed the aiming apparatus through a semi-reflective glass mounted 30 cm above the aiming surface. Positioned 30 cm above the semi-reflective glass was a liquid crystal display monitor (HP Compaq LA1956x, Palo Alto, CA) used to project images onto the glass.

The aiming apparatus was also equipped with a microswitch positioned 32 cm directly in front of the participant. The switch was used as the starting position for the reaching finger. Three piezoelectric buzzers (frequency: 4,000 Hz; KPEG158-P5H, Kingsgate Electric Corp, Taipei-Hsien, Taiwan) were positioned 25 cm to the left of the microswitch and ~ 55 cm away from the participant. The input to the buzzers was adjusted using potentiometers to produce a loud (i.e., 70 dB), a medium (i.e., 58 dB), or a soft (i.e., 48 dB) sound, which identified the target in the auditory-cued conditions (see below).

Three circular indentations were made between the participant and the microswitch, at 15.5 cm, 15 cm, and 16.5 cm from the microswitch (see Fig 1) and -3.5 deg, 0 deg, and 4 deg relative to the participant’s cyclopean eye, respectively. These indentations served as placeholders for the somatosensory targets in the somatosensory target conditions (see below). A black plastic case (40 cm x 9 cm x 3 cm) with an opening towards the participants was used to cover the three indentations and prevent the reaching finger from making contact with the target fingers (i.e., precluding any tactile feedback about endpoint accuracy). The base of each indentation contained the stimulation surface of a solenoid vibrator (type 347–652, Johnson Electric, Shatin, Hong Kong). These solenoid vibrators delivered brief vibrations (50 ms at 80Hz, with a 2 mm amplitude) that indicated the target finger in the vibrotactile-cued somatosensory target condition (see below).

Fixation positions and visual targets (only used in the visual target condition) were projected onto the aiming surface using a custom MATLAB program (The Mathworks Inc., Natick, MA) and Psychtoolbox-3 [18]. Three fixation positions were projected as blue circles, measuring 0.45 deg of visual angle in diameter. They were located 4.5 cm distal to the microswitch, at 0 deg, 10 deg to the right, and 10 deg to the left of the participant’s cyclopean eye. In the visual target condition, targets of 1 deg of visual angle were projected onto the same spatial locations as the indentations utilized for the somatosensory targets (see above).

A white light emitting diode (LED, 4 mm diameter) attached to the participant’s right index finger was visible through the semi-reflective glass and provided visual feedback of the initial fingertip position in the visual-target condition (see below). Also, a black plastic shield was placed beneath the glass surface to occlude vision of the limb during the aiming movement. The position of the occluding surface was adjusted for each participant such that they could see the illuminated LED on their finger when it was at the home position.

Eye positions were monitored with electro-oculography (EOG) (Coulbourn Instruments, Lablinc Inc., Lehigh Valley, PA), sampled at 1000Hz. The position of the index finger was tracked using an electromagnetic sensor secured to the tip of the right index finger (Flock of Birds, Ascension Technology Corp., Burlington, VT), sampled at 100 Hz.

Overall, the participants’ task was to maintain gaze fixation while performing accurate reaching movements with the index finger of the right hand to targets located between the home position and their body. Multiple target locations were included to introduce variable movement parameters to each reaching trajectory. This variability was introduced to reduce the likelihood that participants engaged in sensorimotor processes related to target position encoding before the onset of the imperative stimulus. Participants reached to somatosensory targets in response to either an auditory (AUD-SOMA) or a vibrotactile (TACT-SOMA) cue. To obtain reference values for gaze-dependent reaching errors, participants also performed reaches to visual targets in response to an auditory cue (AUD-VIS). The presentation order of these conditions was counterbalanced across participants.

### Cue-target conditions

In the AUD-VIS condition, participants positioned their left hand in a comfortable position, either on their lap or on the table surface to the left of the aiming apparatus. The trial sequence was initiated once the participant depressed the home position microswitch with their right index finger. This action triggered the illumination of the finger LED and the presentation of the current fixation point (i.e., -10 deg [left], 0 deg [central], or +10 deg [right]). Two seconds later, participants were presented with a soft, medium, or loud sound. For half of the participants, the loud and soft sounds corresponded to the right and left targets, respectively. This correspondence was reversed for the other half of the participants. At movement onset, the finger LED was extinguished, therefore participants only saw the position of their reaching finger relative to the visual target prior to reaching.

The AUD-SOMA condition was similar to the AUD-VIS condition. But instead of visual targets, participants placed the three middle fingers of their left hand (i.e., the ring, middle, and index) into the three circular indentations beneath the plastic casing to serve as the somatosensory targets. The same auditory cues as in the AUD-VIS condition indicated the target finger positions. Participants were instructed to “aim to the perceived position of their fingernail as if it was projected onto the surface of the plastic case”. In contrast to the AUD-VIS condition, participants did not receive visual information about the position of their reaching hand prior to movement initiation.

The TACT-SOMA condition was similar to the AUD-SOMA condition, except that a vibrotactile stimulus indicated the target finger (i.e., index, middle, or ring finger). Similar to the auditory stimulation in the auditory-cued conditions, the tactile stimulation of the target finger occurred 2 s after the participant depressed the microswitch (i.e., the home position).

### Familiarization trials

Before each experimental condition, participants performed 2 sets of familiarization trials. For the AUD-VIS and AUD-SOMA conditions, the first set of familiarization trials consisted of 3 blocks of 3 reaching trials, wherein the same auditory-cue was presented in each block (e.g., 3 trials with the “loud” sound). Subsequently, participants performed 1 block of 15 trials, wherein auditory cues were presented in random order. After each trial in this familiarization block, participants were asked to report which auditory cue had been presented (e.g. “loud”, “medium”, or “soft”) and they were very accurate at distinguishing between the three different levels of sound (mean accuracy: 97%; SD = 0.5%). Participants were also presented with 2 sets of familiarization trials in the TACT-SOMA condition. Auditory cues were replaced by brief tactile stimulation applied to the reaching finger. The participant’s task in the second set of familiarization trials was to report which target finger had been stimulated and participants were all perfectly accurate in this task.

### Experimental trials

In all experimental conditions, participants performed 10 trials in each fixation-target combination (i.e., 3 fixation [-10 deg, 0 deg, +10 deg] by 3 possible targets [left, middle, right], yielding 90 trials per condition) for each of the 3 cue-target conditions (i.e., AUD-VIS, AUD-SOMA, TACT-SOMA) yielding a total of 270 total experimental trials.

### Data analysis and reduction

Only data from movements to the centre target were analyzed because gaze-shifts were equidistant with respect to this target (i.e., 10 deg on either side) and a consistent sound level (i.e., medium sound) was used to signal the target position across conditions. All finger and eye movement recordings were exported to custom software (Analyse, Marcel Kazsap, QC, Canada) and processed offline. Raw EOG traces were used to verify participants eye positions for each reaching conditions and trials wherein the participants failed to maintain their fixation position prior to or during the movement were removed. In addition, trials where participants perceived the incorrect sound, or where reaction times (RT) or movement times were higher or lower than 2.5 times the within condition standard deviation were also excluded from the analyses. Overall, less than 9% of trials (centre target) were excluded (an average of 2.6 ± 1.6 trials per participant). Statistical analyses were performed with the Statistical Package for Social Scientists (version 21: SPSS Inc., Chicago, IL). Post-hoc comparisons were performed using R (version 3.02, R Development Core Team).

Normalized directional reaching errors, reaction times (RTs) and movement times (MTs) were computed to investigate the effect of peripheral fixation on movement performance. Directional reaching error was defined as the angular difference (in degrees) between the home-target position vector and the home-movement end position vector. We used the within-participant mean and standard deviation computed in the centre fixation position to calculate a population z-score value for each trial with left and right gaze fixation. This normalization procedure therefore resulted in values that reflected the participant’s change in performance in response to a peripheral gaze shift relative to their own variability in the central fixation (i.e., no gaze-shift) condition. Negative and positive z-score values indicate movements biased to the left and right of the centre position, respectively.

Reaction time was defined as the time that elapsed between the onset of the go signal (i.e., either the sound or the finger vibration) and the release of the home position microswitch. Movement time was defined as the time that elapsed between the release of the home position microswitch and movement end, which was defined as the first sample at which the instant velocity of the fingertip fell below 30 mm/s.

Normalized directional reaching errors were submitted to a 2-fixation direction (Left, Right) x 3 cue-target condition (AUD-VIS, AUD-SOMA, TACT-SOMA) repeated-measures ANOVA. Reaction times and movement times were submitted to separate 3-fixation direction (Left, Center, Right) x 3 cue-target condition (AUD-VIS, AUD-SOMA, TACT-SOMA) repeated-measures ANOVAs. The alpha level was set at 0.05 for all statistical contrasts, and effect sizes for the ANOVAs were reported as partial eta squared. Post-hoc tests for any significant interactions were completed using pairwise *t*-tests with the Bonferonni correction. Additionally, for directional reaching errors, one-sample *t*-tests were used to examine if peripheral fixation yielded a significant bias (e.g., different than 0 deg) for each fixation direction, effect sizes for the one-sample *t*-tests were reported using Cohen’s d.

## Experiment 1 results

### Normalized directional reaching errors

**Fig 2:**
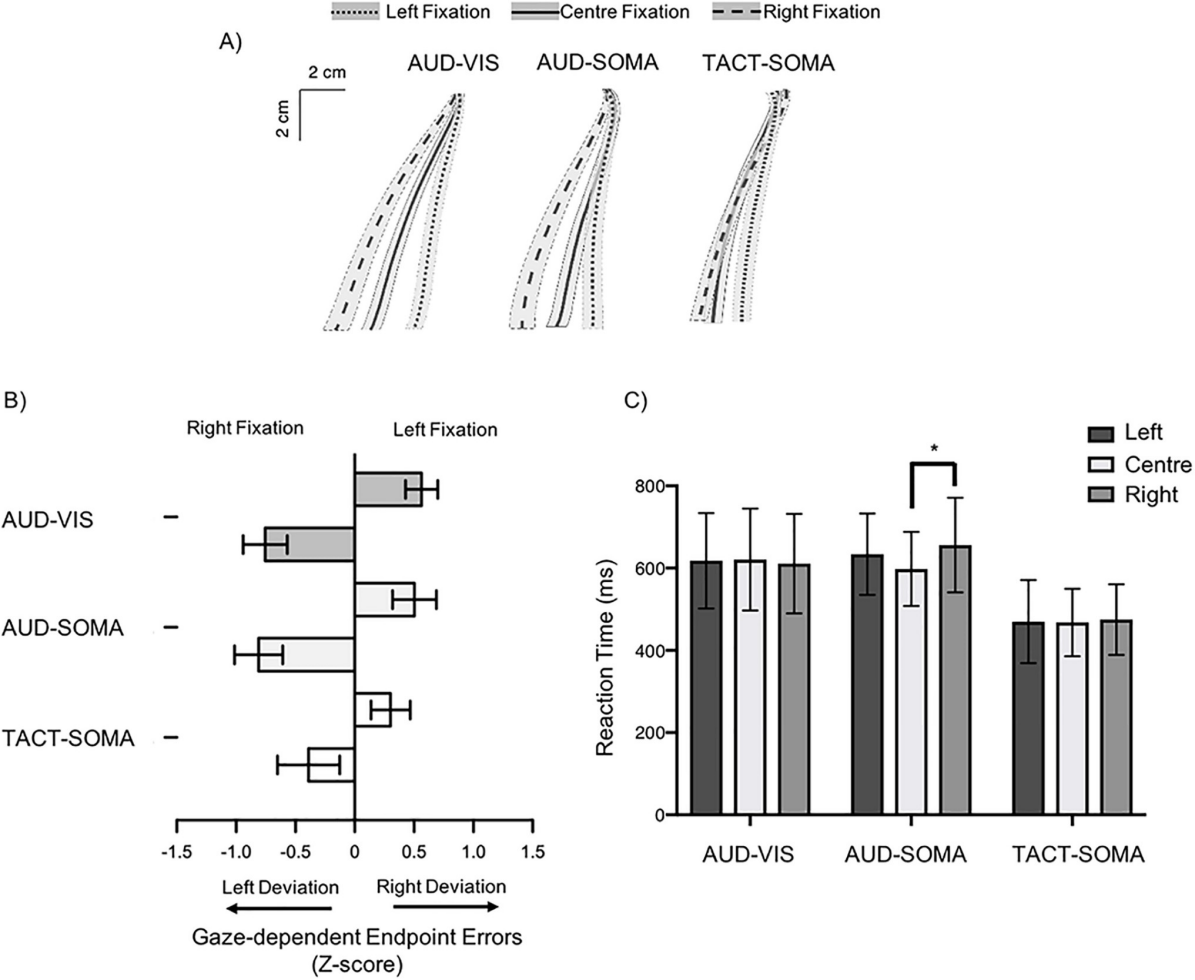
**A)** Averaged reach trajectories for all participants in each cue-target condition (error patches represent the between-participant standard error of the mean). **B)** Average normalized directional error for each cue-target condition (error bars represent the between participant standard error of the mean). Participant’s reaching errors were significantly more influenced by gaze fixation position (i.e., Right Fixation vs. Left Fixation) in the AUD-VIS and AUD–SOMA than in the TACT-SOMA condition. **C)** Mean reaction times in each experimental condition (error bars represent the between-participant standard error of the mean). Reaction times were significantly longer in the AUD-SOMA and AUD-VIS conditions than TACT-SOMA condition. In addition gaze-shifts to the right resulted in longer reaction times in the AUD-SOMA condition.

The ANOVA performed on the directional reaching errors revealed a significant main effect of fixation direction (*F*(1,9) = 64.7, *p* < 0.001 η_P_^2^ = 0.89) and a significant interaction between fixation direction and cue-target condition (*F*(2,18) = 5.1, *p* = 0.018, η_P_^2^ = 0.36). Critically, decomposing the interaction (Bonferonni corrected alpha of p = 0.006) revealed that the differences in gaze-dependent errors for the left and right fixation directions were higher in both the AUD-SOMA (Left: M = 0.50, SD = 0.58; Right: M = -0.81, SD = 0.64 *mean Difference* = 1.31), and AUD-VIS target conditions (Left: M = 0.56, SD = 0.43; Right: M = 0.76, SD = 0.49; *mean difference* = 1.32), compared to the TACT-SOMA condition (Left: M = 0.30, SD = 0.52; Right: M = -0.39, SD = 0.83; *mean difference* = 0.69). Furthermore, one sample *t*-tests revealed that the magnitude of the directional reaching errors in both left and right fixation conditions in the AUD-SOMA (left: *t*(9) = 4.2, *p* = 0.024 *d* = 1.3; right: *t*(9) = -4.1, *p* = 0.003, *d* = 1.3) and AUD-VIS conditions (left: *t*(9) = 2.7, *p* < 0.001, *d* = 0.9; right: *t*(9) = -4.0, *p* < 0.001, *d* = 1.3) were significantly greater than zero. In contrast, normalized directional reaching errors for the left and right fixation directions in the TACT-SOMA condition were not significantly different than zero (left: *t*(9) = 1.8, *p* = 0.099, *d* = 0.6; right: *t*(9) = -1.5, *p* = 0.170). These results provide evidence that gaze-dependent coding was used for sensorimotor transformations when movements were performed to auditory-cued visual and auditory-cued somatosensory targets (see Fig 2A and 2B).

### Reaction times and movement times

Analyses of the RTs revealed a significant effect of cue-target condition (*F*(2,18) = 19.1, *p <* 0.001, η_P_^2^ = 0.68) indicating shorter reaction times in the TACT-SOMA (M = 471 ms SD = 87 ms) condition than in both the AUD-VIS (M = 617 ms, SD = 116 ms) and AUD-SOMA (M = 629 ms, SD = 102 ms) conditions. The ANOVA also revealed a significant condition by fixation interaction (*F*(4, 36) = 3.7, *p* = 0.019, η_P_^2^ = 0.27). The decomposition of the interaction indicated that changes in gaze direction significantly increased reaction times in the AUD-SOMA condition. More specifically, post-hoc tests revealed that, in the AUD-SOMA condition, RTs were significantly longer when participants shifted their gaze to the right (M = 656 ms, SD = 115 ms) compared to when they fixated on the centre position(M = 598 ms, SD = 90 ms). Mean RT was also 36 ms longer in the left fixation condition (M = 634 ms, SD = 99 ms) than in the centre fixation condition, but this increase of RT fell short of statistical significance (*p* = 0.062). For both the AUD-VIS and TACT-SOMA conditions, the RTs were not significantly different between any of the fixation conditions. In addition, no significant differences in RT were found between the centre fixation conditions between the AUD-VIS and AUD-SOMA conditions. Finally, the analyses of MTs yielded no significant effects of fixation direction or cue-target condition, and no significant interaction between these factors (*F*s < 1.6, *p*s > 0.16).

### Experiment 1 discussion

In Experiment 1, participants performed reaching movements to one of three somatosensory target positions cued by either an auditory or a vibrotactile stimulus. To provide a comparison for gaze-dependent reaching errors, participants also performed movements to visual targets cued by an auditory stimulus. Overall, participants exhibited larger gaze-dependent endpoint errors when reaching to auditory-cued somatosensory targets compared to vibrotactile-cued somatosensory targets. Also, gaze-dependent reaching endpoint errors for auditory-cued somatosensory targets were no different than those observed for reaches to auditory-cued visual targets. Taken together, these findings suggest that the modality of the sensory cue indicating the position of the somatosensory targets had an impact on the type of body representation that was used to plan movements.

Participants exhibited larger gaze-dependent reaching errors in both auditory-cued target conditions compared to the vibrotactile-cued somatosensory target condition. These results are congruent with earlier studies reporting that movements to somatosensory targets were planned in gaze-independent coordinates if the eyes and target positions remained stable prior to movement onset [13, 14]. One explanation for these findings is that the vibrotactile cue facilitated the encoding of target position in somatosensory coordinates. Cutaneous and proprioceptive inputs project to different areas (cutaneous: areas 3b and 1; proprioceptive: areas 3a and 2) of the post-central cortex [19, 20], and recent investigations have shown that there are reciprocal connections that enable these areas to respond to both types of somatosensory stimuli (see [21]). Given the close link between tactile stimulation and the somatosensory body representation (see [22]), the integration of spatially congruent somatosensory inputs in our TACT-SOMA condition may have favoured the use of an interoceptive body representation for the encoding of the finger target position when planning the reaching vector.

Consistent with the gaze-dependent biases found in the AUD-SOMA condition, previous studies have also found that somatosensory targets can also be represented in visual coordinates [15, 23, 24]. In contrast to the TACT-SOMA condition, the absence of tactile stimulation of the target finger in the AUD-SOMA condition might have precluded the encoding of the target finger position in gaze-independent somatosensory coordinates. Thus, movements prompted by exogenous auditory cues, likely employed an exteroceptive representation of the body for movement planning processes. These findings are consistent with previous studies that showed that if an exteroceptive representation is used to define a body position, the location of this position is more biased by environmental visual information [25].

In the present study, reaction times were shorter when initiating movements to vibrotactile-cued somatosensory targets than auditory-cued somatosensory and visual targets. This result is consistent with studies showing that participants are faster when reacting to somatosensory stimuli than visual or auditory stimuli [26–28]. More relevant to the present study, reaction times significantly increased when participants gazed at the right fixation position when planning movements to auditory-cued somatosensory targets. A tendency towards an increase in reaction time was also found during left gaze fixation. This finding might suggest that the sensorimotor transformations required to use an exteroceptive body representation when reaching to somatosensory targets, in conditions with peripheral fixation, involve a rotation of the movement vector (see [29, 30]).

Moreover, the absence of significant differences in gaze-dependent reaching endpoint errors between the AUD-VIS and AUD-SOMA conditions also suggests that an exteroceptive representation was used to plan movements to somatosensory targets in the AUD-SOMA condition. Thus, it is possible that the initial movement vector for both movements to auditory-cued visual targets and auditory-cued somatosensory targets were defined in visual coordinates. Planning a movement to an auditory-cued somatosensory target, defined in visual coordinates, would likely require a remapping of somatosensory target positions prior to the computation of the movement vector (i.e., a reference frame conversion, see [31]). This remapping of the somatosensory target positions would also likely require greater visual processing in areas involved in visuomotor transformations and gaze-dependent movement coding [32–35], compared to conditions where no conversion of somatosensory information is required (e.g., reaches to auditory-cued visual targets). A second experiment was designed to test this prediction.

### Experiment 2 introduction

In Experiment 1, participants showed gaze-dependent endpoint errors when reaching to somatosensory targets cued by auditory stimuli. This result was attributed to the use of an exteroceptive, visually-based, body representation when reaching to somatosensory targets. The purpose of Experiment 2 was to examine the neural mechanisms associated with the gaze-dependent encoding of somatosensory target positions during movement planning.

Although behavioural evidence for the remapping of somatosensory targets onto a gaze-dependent reference frame has been reported (e.g. [13–15]), the neural processes underlying these sensorimotor transformations remain largely unknown. Previous studies examining motor planning have implicated the occipito-parietal network in the gaze-dependent coding of effector and target positions [32, 33, 35]. Moreover, studies on autotopagnosia have shown that the inability to use the exteroceptive body representation is linked to damage to the left posterior parietal and parietal-occipital cortices [2–6, 36]. Given these findings, we hypothesized that there would be greater activation in parieto-occipital networks when preparing movements to somatosensory targets encoded in visual coordinates as compared to external visual targets. Note that for the external visual targets, no remapping is required as both hand and target positions are already presented in an extrinsic coordinate system [37–39].

To test these predictions, the cortical response to a task-irrelevant visual stimulus (i.e., the visual-evoked potential or VEP) was measured as participants planned movements to both auditory-cued visual and auditory-cued somatosensory targets. Because baseline neural activity in the extrastriate cortex is a marker of visual processing, and the amplitude of visually-evoked responses increases along with baseline activity [40, 41], comparing changes in VEP amplitudes should provide a good proxy for the engagement of visual and visuomotor cortical networks.

## Experiment 2 methods

### Participants

Ten participants (5 female, mean age: 26 ± 4.7; range 20–35), who did not participate in Experiment 1, were recruited for Experiment 2. All participants self-reported being right-handed and had normal or corrected-to-normal vision. Informed consent was obtained prior to data collection and a local ethics committee at Aix-Marseille University approved all procedures. The protocols and procedures employed in this experiment were in accordance with the 1964 Declaration of Helsinki. In total, the experiment took place over 2 sessions lasting 1.5 to 2 hours each. There was at least 1 but no more than 10 days between sessions (average = 4 ± 3.5 days).

### Apparatus

**Fig 3:**
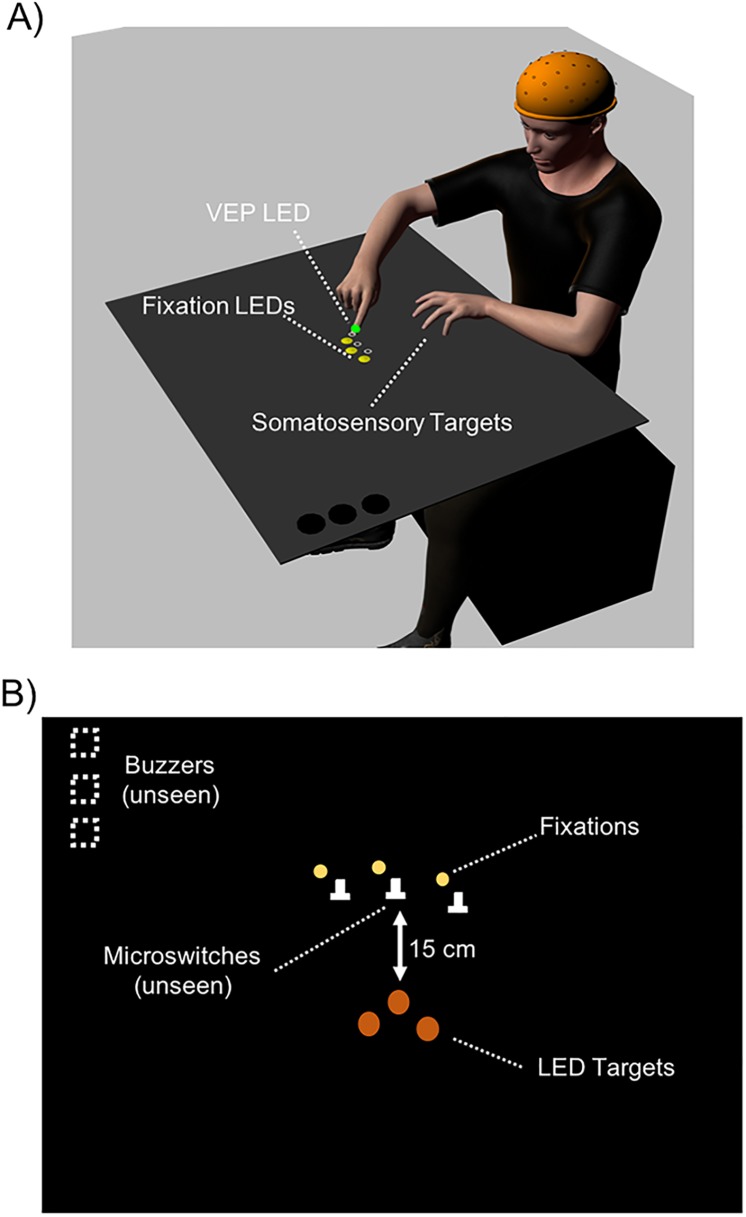
A) A depiction of the experimental apparatus used in Experiment 2 (not to scale). Participants fixated on one of three fixation locations and began their movements from the associated home position. As in Experiment 1, in both the AUD-VIS and AUD-SOMA conditions, piezoelectric buzzers indicated the targets and provided the imperative signal to initiate the reaching movement. In the AUD-SOMA condition, participants performed movements to either the index, middle, or ring finger of their non-reaching limb. In the AUD-VIS condition, participants performed movements to one of the three visual targets. B) A depiction of the aiming surface in the AUD-VIS condition (not to scale). There were three possible home positions such that the location of the task-irrelevant visual stimulus (the evoked potential stimulus) was in the same retinal location relative to the fixation position.

To accommodate the use of electroencephalography (EEG) and reduce electrical noise, all visual stimuli were generated using LEDs positioned on the aiming surface (see Fig 3) instead of the LCD monitor used in Experiment 1. Two additional microswitches and 3 yellow LEDs were added to the aiming surface. Each additional microswitch served as a different possible starting position. Each LED was placed 0.5 cm distal and 0.5 cm to the left of each microswitch and served as a possible fixation location (see procedures below). Thus, when participants fixated on any of the LEDs and placed their finger on the corresponding microswitch, the finger was positioned in the same relative retinal location, in the lower right visual field.

Three orange LEDs were placed at the same positions as the circular indentations that served as the somatosensory target position in Experiment 1. These LEDs were used as visual targets in the AUD-VIS condition. To prevent participants from receiving tactile feedback when reaching to visual targets, a thin piece (39 cm x 9 cm x ~0.3 cm) of translucent laminated Bristol board was placed over the LEDs. In addition, the participant’s right index finger was equipped with two LEDs: the same white LED that was used in Experiment 1 to indicate the initial position of the pointing finger, and a green LED (width 5mm, beam angle 40°, luminance 60000 mcd, 22.7 lm) attached to the fingernail which was used to generate the stimuli for the VEPs.

### Trial procedures

In contrast to Experiment 1, no gaze-shift stimulus was presented prior to the reaching movement. As the purpose of Experiment 2 was to evaluate the differences in cortical network activation underlying reaches to auditory-cued somatosensory and visual targets, it was critical that visual stimulus was presented in the same retinal location prior to the start of the reaching movement.

Each trial began when one of the three possible fixation positions was illuminated. Participants looked at the fixation LED and placed their right index finger onto the corresponding microswitch (see Fig 3B). As in Experiment 1, a soft, medium, or loud sound was presented 2 seconds after the participant placed their finger on the microswitch. That auditory cue indicated either the somatosensory or visual target and served as the imperative signal to begin the pointing movement. Then, 100 ms after the presentation of the auditory cue, during the participant’s reaction time (i.e., movement planning), the green LED located on the pointing finger generated a 50 ms flash. This presentation time was chosen based on previous studies which showed significant modulations in evoked responses [42] and reach-related activity [43] for both movements to somatosensory and visual targets (i.e., between 100–150 ms after the go signal). Participants were instructed to reach to the target as precisely as possible and to stay at the target location until the next fixation point was illuminated.

Only one cue-target condition was used per experimental session (AUD-VIS or AUD-SOMA), and the presentation order of the target modality was counterbalanced across participants. As in Experiment 1, the sound-target mapping was counterbalanced between participants and remained the same for both experimental sessions. Movements were also conducted in darkness such that participants had no visual feedback of the reaching limb during the movements.

## Cue-target conditions

In the AUD-VIS condition, when participants placed their finger on the home position microswitch, the finger and all target LEDs were illuminated. When participants released the microswitch to begin their reaching movement, the finger LED was extinguished, but all target LEDs remained illuminated throughout the trajectory. Therefore, participants could get terminal feedback about their movement accuracy through the reflection of the illuminated targets on their reaching finger. In contrast to Experiment 1 where gaze-dependent reaching endpoint errors were assessed, the difference in endpoint error between conditions was not relevant to the purpose of Experiment 2; thus, participants were allowed to have terminal feedback in both conditions.

In the AUD-SOMA condition, participants performed reaches to the fingernail of one of the three middle fingers on their left hand. As in the Experiment 1, participants did not receive visual feedback of their reaching finger during movement planning. Participants also had terminal feedback in the AUD-SOMA condition as they made physical contact with the target finger.

### Control condition

The magnitude of VEPs varies considerably between participants (e.g., due to difference in impedance and thickness of the skull; see [44]). For this reason, a series of trials were performed in a control condition before both experimental sessions in order to normalize VEPs for comparison across participants and sessions. The main difference between the control and experimental trials was the absence of reaching movements.

For the AUD-VIS control condition, when the microswitch was depressed, the finger and target LEDs were illuminated. Two seconds later, one of the three sounds was presented (i.e., soft, medium or loud). This sound was followed, 100 ms later, by the 50 ms flash of the green LED. Participants remained on the home position until the fixation stimulus was turned off (i.e., ~3s after it was turned on). The same trial procedure was used for the AUD-SOMA control condition, except that the participants placed the middle fingers of their right hand on the somatosensory target position and the reaching finger and target LEDs were not illuminated. As in the AUD-VIS condition, the green LED was flashed (for 50 ms) 100 ms after the presentation of the auditory stimulus.

### Data recording

EEG data were recorded continuously from 64 pre-amplified Ag-AgCl electrodes (ActiveTwo, Biosemi, Amsterdam, Netherlands) embedded in an elastic cap mapped to the extended 10–20 system. Two electrodes, a Common Mode Sense (CMS) active electrode and a Driven Right Leg (DRL) passive electrode specific to the Biosemi system, served as a feedback loop driving the average potential of the measured signal to levels as close as possible to the analog-to-digital converter reference voltage. EOG was recorded bipolarly with surface electrodes placed near both outer canthi as well as under and above the orbit of the right eye. EOG recordings were used to identify ocular artefacts (see below) and also allowed the experimenters to verify that participants fixated on the fixation LEDs during movement planning and execution. The EEG and EOG signals were digitized (sampling rate 1,024 Hz, DC, 268 Hz, 3 dB/octave) and band-pass filtered (0.1–45 Hz, notch filter applied at 50Hz, 12 dB/octave). As in Experiment 1, kinematic data of the reaching finger were recorded by tracking the positions of an electromagnetic sensor fixed on the top of the index finger and sampled at a rate of 100 Hz.

### Data analysis

VEPs were obtained by averaging the time-locked EEG signals with respect to the onset of the 50 ms green flash (-200 to +300 ms). These epochs were averaged for each participant and for each condition. The mean amplitude of the -200 ms to -5 ms segment of each epoch served as the pre-stimulus baseline. The monopolar recordings were referenced to the average of the right and left mastoid electrodes. Recordings were visually inspected and epochs with eye movements or artefacts were rejected. The average number of traces used for each condition varied from 172 to 179 (out of a possible 180), and there were no significant differences between the amount of trials rejected between conditions as revealed by a 2 phase (control, experimental) by 2 cue-target condition (AUD-VIS and AUD-SOMA) repeated-measures ANOVA. The result of this analysis indicates that the signal-to-noise ratio was not different across conditions.

A current source density (CSD) analysis was employed to increase the spatial resolution of EEG signals [45–47]. The signal was interpolated with a spherical spline interpolation procedure to compute the second-order derivatives in two-dimensional space (order of splices: 3; maximal degree of Legendre polynomials: 10; approximation parameter lambda; 1.0e-005: see [45]). CSD measurements represent a reference-free estimate of activity at each electrode and are less affected by far-field generators than monopolar recordings [46, 48, 49]. Thus, CSD analyses tend to yield measures that better reflect the underlying cortical activities and local sources [50, 51].

CSD-VEPs that were computed from the left posterior-occipital and left occipital-parietal electrodes sites (O1, PO3, PO7) were used for the main analyses. These were chosen based on previous studies which implicated the underlying cortical areas in sensorimotor transformation processes and the use of the exteroceptive body representation [33, 35,52–55].

**Fig 4:**
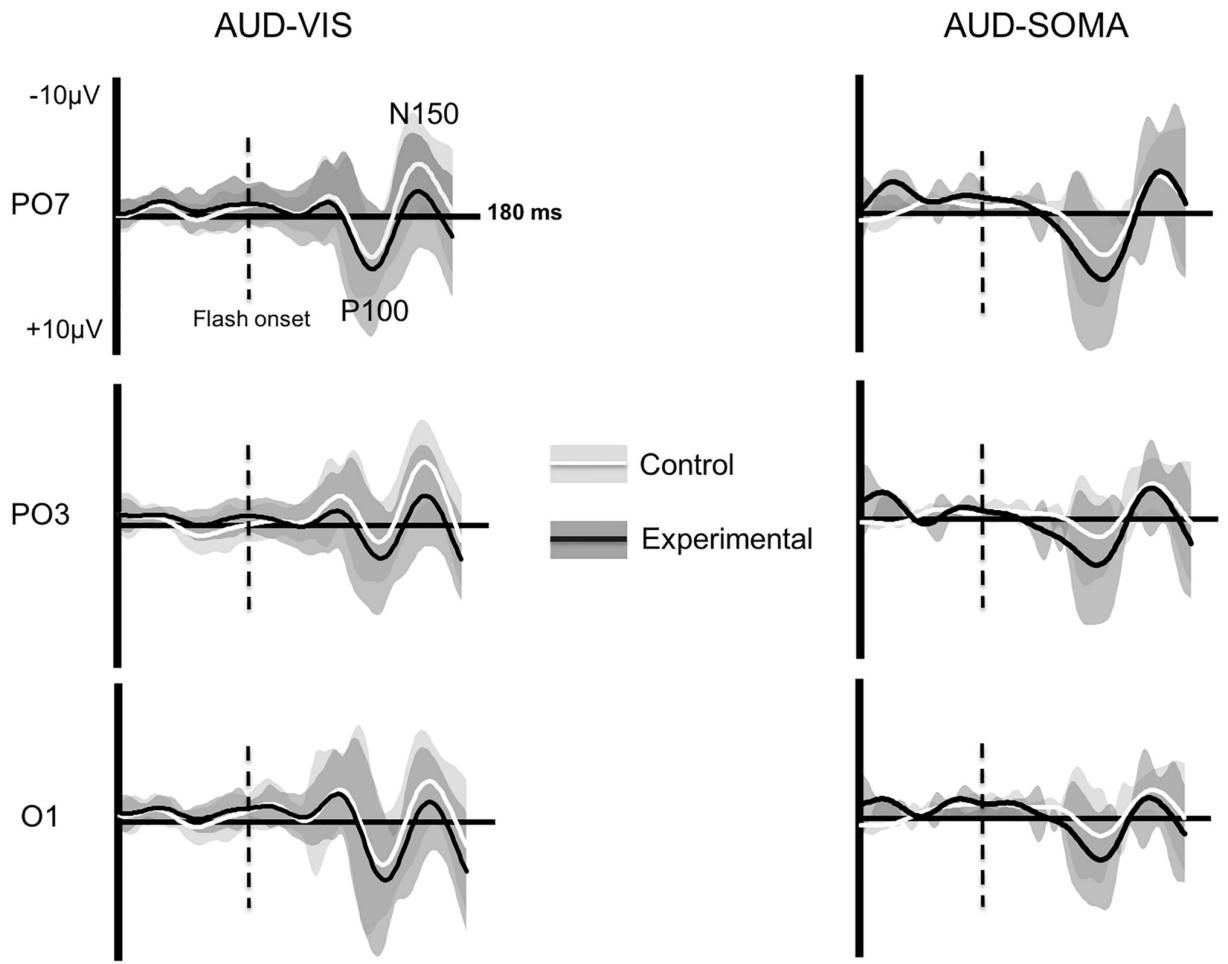
Grand average VEPs for each electrode in the AUD-VIS and AUD-SOMA conditions (error patches represent the between-participant standard deviation).

CSD-VEPs were assessed, for each electrode, by measuring the peak-to-peak amplitude between the first positive (~100 ms) and negative (~150 ms) deflections after stimulus onset that could be identified in all participants and conditions (see Fig 4). The latencies of these deflections are reported for each electrode in Table 1. The amplitude of the P100-N150 (hereafter referred to as the VEP) was expressed as the ratio of the CSD-VEP amplitude measured in the Control and Experimental conditions (CSD-VEP ratio = log2[CSD-VEP experimental conditions/CSD-VEP control conditions]). CSD-VEPs were computed using a log_2_ transformation to account for the nonlinearity of ratios. Normalized CSD-VEP amplitudes were then used for statistical contrasts. An increase in normalized CSD-VEP amplitude was considered indicative of additional visual processing in the experimental condition relative to the control condition during the early stages of movement planning.

**Table 1:** Mean (and standard deviation) latencies (in ms) for the peaks used in the CSD- VEP calculation.

	AUD-VIS				AUD-SOMA			
	Control		Experimental		Control		Experimental	
Electrodes	P100	N150	P100	N150	P100	N150	P100	N150
PO3	111 (23)	151 (26)	102 (16)	147 (14)	103 (18)	145 (10)	107 (14)	147 (12)
PO7	103 (7)	144 (13)	101 (14)	156 (16)	106 (13)	151 (14)	109 (8)	152 (17)
O1	101 (15)	140 (13)	104 (10)	153 (16)	112 (13)	148 (12)	106 (11)	143 (15)

The latencies of the CSD-VEPs were analyzed using a 2-phase (control, experimental) by 2-condition (AUD-VIS, AUD-SOMA) repeated-measures ANOVA for each electrode of interest. The analysis of latencies did not reveal any significant main effects or interactions between fixation direction and cue-target conditions for either the P100 or N150 component for any electrode (ps > 0.054, Fs < 4.9).

The neural sources of the P100-N150 in all experimental and control conditions were estimated using minimum norm technique as implemented in the Brainstorm software suite [56]. To resolve the inverse problem and estimate the cortical sources of the VEPs, data were imported from all sensors, processed, and averaged for each participant, condition, and electrode. The forward model was computed for each condition using a symmetric boundary element method (BEM, [57]) on the anatomical MRI brain template from the Montreal Neurological Institute (MNI Colin27). Sources of the averaged absolute activity were estimated using the dynamic statistical parametric maps (dSPM) technique [58].

Both behavioural temporal measures (i.e., movement time and reaction time) as well as normalized CSD-VEP amplitudes were submitted to paired samples t-tests. Effects sizes were reported with Cohen’s d_z_.

## Experiment 2 results

### Behavioural results

Overall, there were no significant differences between any behavioral variables related to the temporal aspects of movement performance between the AUD-VIS and AUD-SOMA conditions. Paired samples t-tests did not reveal significant differences in movement times (overall mean 737 ms ± 136 ms; *t*(9) = -0.1, *p* = 0.894, *d*_*z*_ = *0*.*04*). There were also no statistically significant differences in reaction times (overall mean 498 ms ± 67 ms; *t*(9) = -2.2, *p* = 0.063, *d*_*z*_ = *0*.*7)*. The latter observation suggests that, despite a tendency towards greater RT in the AUD-SOMA condition, the timing of the VEP stimulus presentation relative to the motor planning process was not significantly different between both conditions.

### CSD-VEPs

**Fig 5:**
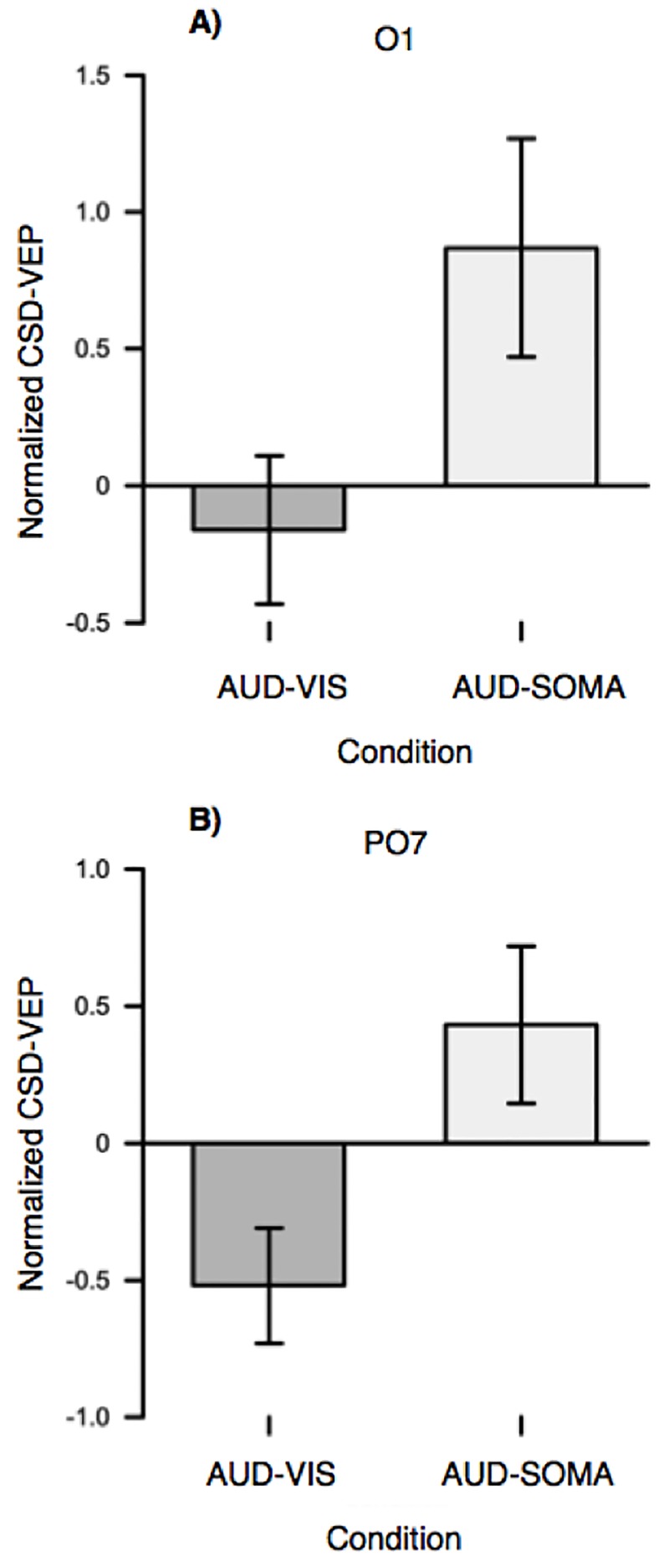
CSD normalized VEPs for the occipital (O1) and occipito-parietal (PO7) electrodes (error bars represent the standard error of the mean). For both electrodes, CSD- VEPs were significantly larger in the AUD-SOMA compared to the AUD-VIS condition.

Evidence for an increase in visual information processing during movement planning was found for reaches to auditory-cued somatosensory targets compared to auditory-cued visual targets in both the occipital (O1: see Fig 5A) and occipital-parietal (Po7: Fig 5B) electrodes. At the O1 electrode, normalized (log2) VEP amplitudes in the AUD-SOMA condition (*M* = 0.87, *SD* = 1.2) were significantly larger (*t*(9) = 3.2, *p* = 0.053, *d*_*z*_ = 0.7) than those observed in the AUD-VIS condition (*M* = -0.16, *SD* = 0.85). VEP amplitude differences were also found at the PO7 electrode (*t*(9) = 3.2, *p* = 0.017, *d*_*z*_ = 1.0), as the amplitude of the P100-N150 component was larger when planning movements in the AUD-SOMA condition (*M* = 0.43, *SD* = 0.90) as compared to the AUD-VIS condition (*M* = -0.51, *SD* = 0.66). In contrast, there were no significant CSD-VEP differences at the PO3 electrode (*t*(9) = -0.39, *p* = 0.702, *d*_*z*_ = 0.13).

**Fig 6:**
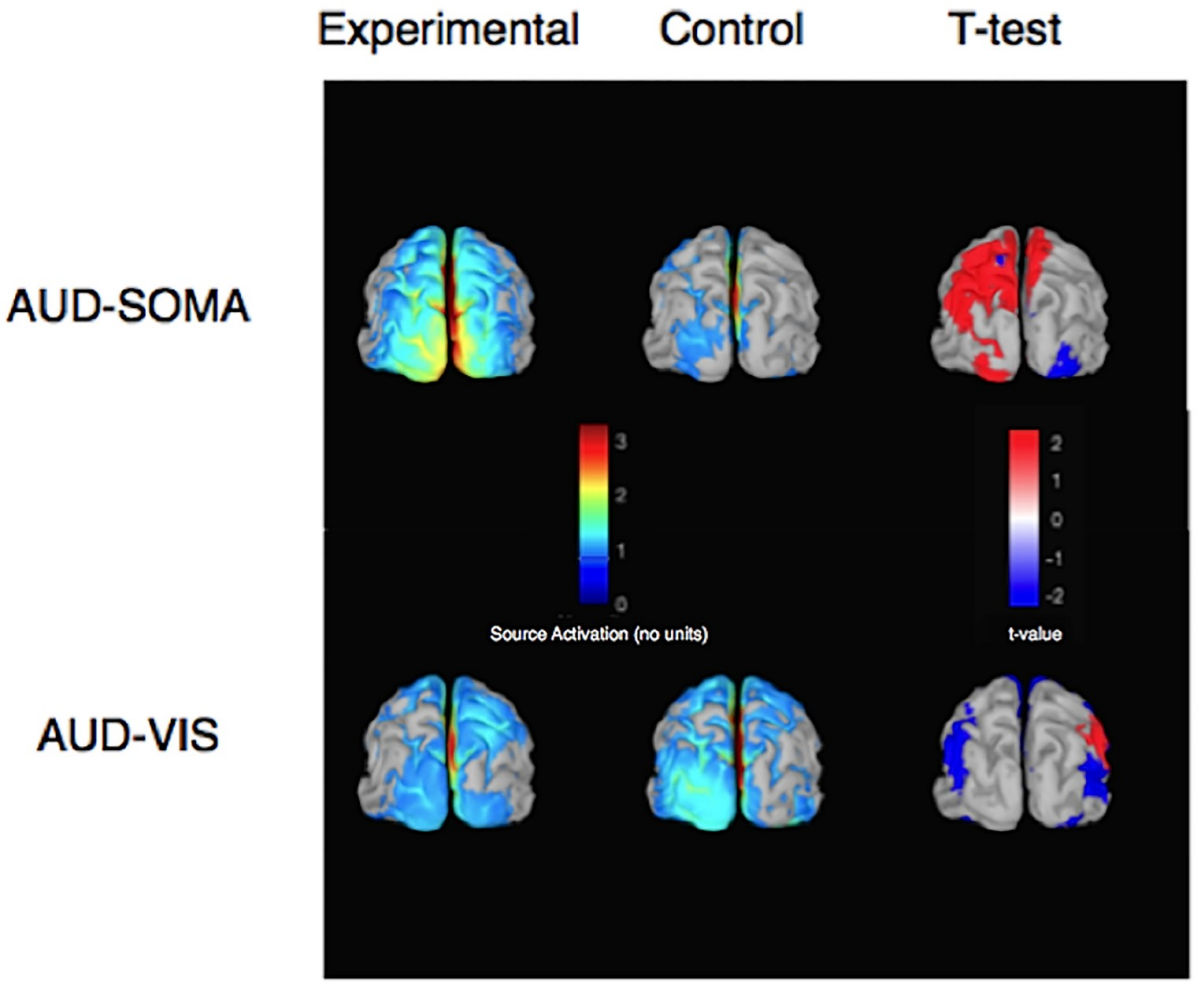
Grand average source activity for each condition between 100 ms and 150 ms after stimulus onset (i.e., source activity corresponding to the P100 –N150 component of the visual evoked potential). Source activity (color maps reveal activation levels) was localized in parietal and occipital areas in both experimental and control conditions. Statistical contrasts (paired-samples t-tests, alpha set to 0.05, t-value map indicates direction of effects) revealed significantly more activity in the left parietal and parieto-occipital regions (as indicated by the shade of red) for the AUD-SOMA experimental condition compared to the AUD-SOMA control condition.

Fig 6 shows the average source activity between 100 and 150 ms (i.e., between the peaks of the P100-N150 used for the VEP calculation) projected on a cortical template for all experimental and control conditions in both the AUD-SOMA and AUD-VIS conditions. The source analyses revealed a greater response to the flash in left occipito-parietal areas in the AUD-SOMA compared to the AUD-VIS condition. Interestingly, contrasting the mean activity between the AUD-SOMA condition and its control condition revealed that movement planning led to significantly greater visual-related activation of the left occipital and posterior parietal cortices. Conversely, no significant increases in activity were found in these regions when contrasting the AUD-VIS condition with its control condition.

### Experiment 2 discussion

In Experiment 2, the cortical response to a task-irrelevant visual stimulus was measured as participants performed reaches to both auditory-cued visual and auditory-cued somatosensory targets. Overall, the results of both the CSD and source analyses revealed that participants had a greater response to visual inputs in when reaching to auditory-cued somatosensory vs. visual targets. These findings are consistent with the hypothesis that visual remapping of somatosensory target positions employs cortical networks associated with visual processing and visuomotor transformations.

The increased sensitivity of occipital networks to visual inputs may be linked to a re-weighting of sensory information prior to engaging in higher-order computations. Occipital cortical areas have been implicated in early multisensory integration processes (see [59] for a recent review) and the increased activation observed in the current study could be indicative of the role of these networks in the encoding of body positions in visual coordinates. The suggestion that left-occipital activity could be associated with the visual coding of somatosensory effector and target positions is supported by neuroimaging studies that revealed the presence of hand-selective cells of the lateral occipital cortex are lateralized to the left hemisphere [60, 61].

In addition to enhanced activity in occipital areas, source analyses revealed an increase in neural activity in the left PPC when participants planned movements to auditory-cued somatosensory targets. This result supports the findings of several studies showing that the left PPC is associated with the utilization of the exteroceptive body representation [2–4,6, 36, 54]. Previous research has also revealed that PPC neurons are responsible for the gaze-dependent encoding of the hand and target positions [35, 38, 62, 63]. Although these studies have mostly used visual targets and visible body positions, there is some evidence that gaze-dependent coding can occur in conditions without visual inputs [34, 35,64]. In a PET functional brain imaging study, Darling et al. [34] found an increase in neural activation in the occipital and posterior parietal lobes when participants reached to a memorized somatosensory target (i.e., the previous position of their unseen hand before passive displacement). Based on these findings, the authors suggested that networks employed to guide reaches to visual targets were also used when reaching to memorized somatosensory targets. Our findings therefore build on those of Darling et al. [34] by showing that visual processes may also be involved when planning movements to the actual, non-memorized, position of somatosensory targets.

Previous studies have shown that, in some cases, visual information is attenuated (i.e., gated, see [65]) during movement planning to somatosensory targets [42, 66–68]. Evidence from both behavioural and neurophysiological experiments have argued that, when target locations are encoded in somatosensory coordinates (e.g., fingers of the non-reaching hand), visual information about the reaching limb does not contribute to the same extent to motor planning processes [68]. Although there is substantial evidence that sensorimotor transformation networks exist to support the direct conversion of somatosensory information into a movement vector, there is conflicting evidence about the contexts wherein these networks are employed [38, 43, 69–71]. Based on our results, we suggest that one contextual factor that could influence (or preclude) the use of somatosensory-based sensorimotor transformations is the body representation used to define target locations during motor planning.

It should be noted that the results of the present study do not necessarily support a disengagement of the occipital cortex and PPC when planning movements to auditory-cued visual targets. Rather, the results of the present study suggest that planning movements when vision of the reaching hand and target is available does not require additional processing from areas sensitive to visual inputs compared to the control condition, at least at the time when the visual probe was presented (i.e., 100 ms after target cue presentation).

## Conclusion

In summary, we found that the sensory modality employed to identify the position of a somatosensory target impacts the body representation used for movement planning processes. Preparing reaching movements towards auditory-cued somatosensory targets prompted the use of an exteroceptive representation of the body. By measuring the cortical response to a visual stimulus presented during movement planning, we also found that the additional sensorimotor transformation processes involved in the visual remapping of somatosensory target positions were associated with increased visual processing in occipital and posterior parietal areas. Taken together, the findings of the present study suggest that the sensorimotor transformation processes underlying movements to somatosensory targets derived using an exteroceptive body representation recruits visual and visuomotor cortical networks.
